# The Effect of Infiltration Temperature on the Microstructure and Magnetic Levitation Force of Single-Domain YBa_2_Cu_3_O_7-x_ Bulk Superconductors Grown by a Modified Y+011 IG Method

**DOI:** 10.3390/nano15010021

**Published:** 2024-12-27

**Authors:** Nuerseman Maimaiti, Abulizi Abulaiti, Wanmin Yang

**Affiliations:** 1 School of Traffic and Transportation Engineering, Xinjiang University, Urumqi 830017, China; 2 School of Electrical Engineering, Xinjiang University, Urumqi 830017, China; 3 School of Physics and Information Technology, Shaanxi Normal University, Xi’an 710062, China

**Keywords:** Y-123, RE + 011 IG, high-temperature infiltration, levitation force

## Abstract

During the preparation of single-domain (S-D) REBa_2_Cu_3_O_7-x_ (RE-123) superconducting bulks, the seed crystals can serve as templates for crystal growth, guiding the newly formed crystals to grow in a specific direction, thereby ensuring the consistency of the crystal orientation within the sample. However, the infiltration temperature is typically restricted to approximately 1050 °C when employing NdBa_2_Cu_3_O_7-x_ (Nd-123) crystal seeds in the traditional top-seeded infiltration growth (TSIG) technique for producing single-domain Y-123 bulk superconductors. In the present study, to overcome the temperature limitations of the heat treatment process, the optimized Y_2_O_3_ +011 IG (011 refers to BaCuO_2_ powder) method was employed to fabricate a group of single-domain Y-123 bulks with a high-temperature infiltration (1000–1300 °C). The reason for the differences in the superconducting properties between the different samples was analyzed by studying the relationship between the microstructure of the infiltrated pellet and the final Y-123 sample. The research findings were as follows: (1) when the infiltration temperature exceeded 1150 °C, the successful preparation of single-domain YBa_2_Cu_3_O_7-x_ (Y-123) bulks became unattainable due to the coarsening or melting decomposition of the Y_2_BaCuO_5_ (Y-211) phase according to the SEM–EDS analysis; (2) the content of the Y-211 phase within the Y-123 matrix was approximately 40.8%, 37.2%, 32.7%, 30.5%, and 46.4% for the different final samples; (3) with an increasing infiltration temperature, the magnetic levitation forces exhibited an initial increase followed by a subsequent decline. The maximum levitation force of 47.1 N at 77 K was reached in the sample S3 infiltrated at 1100 °C.

## 1. Introduction

Single-domain (S-D) bulk REBa_2_Cu_3_O_7-x_ (REBCO, where RE represents the following: Yttrium (Y), Gadolinium (Gd), Neodymium (Nd), Samarium (Sm), or other rare earth elements) superconductors have already become a key material in a variety of superconducting engineering applications [1,2,3,4,5,6,7] owing to their strong trapped field properties at low temperatures and their high Critical Current Density (J_c_), etc. As reported, the trapped field of a Y-123 bulk (26.5 mm in diameter) has reached 17.24 T at 29 K [2], and more recently, a GdBCO stack (24.2 mm in diameter) successfully trapped a field of 17.6 T at 26 K [1]. Nb- or Mo-rich NEG-BaCuO nanoparticles (~10 nm) achieved 100 kA/cm^2^ at 90.2 K [8], and the irreversibility field of NEG-123 has exceeded 14 T at 77 K [9].

To date, the top-seeded melt growth (TSMG) method [10,11] and the TSIG [12,13,14] process have been widely used to fabricate the S-D REBCO bulks. Among them, the TSIG process has overcome many of the limitations associated with the traditional TSMG technique, including reducing the pores and macro cracks, minimizing the significant loss of Ba–Cu–O liquid phases, achieving a more uniform distribution of RE-211 particles and addressing the shrinkage of the final samples [15,16,17,18]. While employing the TSIG process, the entire growth process can be viewed as two key stages, i.e., the infiltration of the liquid phase stage (abbreviated as IS in the following text) and the growth of the crystal stage (abbreviated as GS). In the course of the IS, the copper-rich Ba–Cu–O infiltrates into the RE-211 preform pellets; on the other hand, during the GS, a peritectic reaction takes place between the Ba–Cu–O and RE-211 phases, resulting in the formation of the RE-123 phase [19]. However, changes of the melt process parameters in the IS and/or GS may lead to large superconducting performance differences in the final sample. For example, the significance of factors such as the preform porosity or the density of the preform pellets in the TSIG process has been emphasized in Refs [20,21].

Namburi et al. [22] have reported a novel two-step method based on the TSIG process for preparing the S-D Y-123 bulks. In the above studies, even though the S-D Y-123 samples had been prepared successfully, the temperatures chosen for the high-temperature infiltration were only 950, 1050, and 1150 °C. Recently, Yang et al. [23] infiltrated the liquid phase at a high temperature (1100 °C) while employing the RE + 011 IG technique (011 refers to BaCuO_2_ powder), successfully fabricating the high-quality S-D Y-123 bulks. Moreover, in reference [24], Yang et al. also studied the impact of the liquid phase infiltration quality on the S-D GdBCO bulks at various infiltration temperatures (T*_i_*s) ranging from 900 °C to 1040 °C. Their TGA results indicated that the growth and decomposition of Y-123 (consists of Y_2_O_3_ + 10BaCuO_2_ + 6CuO) were approximately 960 °C and 1015 °C, respectively. In addition, the onset temperature for the liquid phase infiltration into the SP pellet is approximately 920 °C; the liquid phase fully infiltrates to the top of the SP pellet at approximately 1020 °C; as the T_i_ is further increased, the volume fraction (V_f_) of the liquid phase that infiltrates into the SP pellet increases. However, to date, there has been no systematic study on whether the infiltration of the liquid phase at higher temperatures can help to improve the superconducting performances of S-D RE-123 samples; hence, it is crucial to determine the optimal T_i_ while using the TSIG technique.

This study demonstrates the production of a group of S-D Y-123 bulk superconductors (φ = 20 mm) through the utilization of high-temperature infiltration employing an enhanced Y + 011 IG process. The T*_i_* was set at 1000 °C, 1050 °C, 1100 °C, 1150 °C, 1200 °C, 1250 °C, and 1300 °C during the IS. At the same time, the relationship between the T*_i_* and the microstructure of the infiltrated pellets and the well-grown Y-123 bulk samples, as well as the magnetic performance of the final sample, was investigated in detail.

## 2. Experimental Sections

### 2.1. Preparation of Precursor Powders

The single-phase 011 powder was synthesized using a traditional solid-state reaction technique in ambient air, employing the purity powders of BaCO_3_ and CuO, which were weighed in the molar ratio of BaCO_3_:CuO = 1:1 and calcined at 900 °C–910 °C for 24 h after initial grinding by a ball-milling machine. To ensure the purity of 011 powder, the same operation was repeated three times. Then, 011 and Y_2_O_3_ were thoroughly combined in a molar ratio of 1.2:1 [25]. The mixture was batch-pressed into cylindrical pellets (φ = 20 mm) weighing 12 g to serve as SP pellets. Next, 011, CuO, and Y_2_O_3_ were thoroughly blended in a molar ratio of 10:6:1 [26]. The 20 g mixture was pressed into cylindrical pellets (φ = 30 mm) for use as liquid phase pellets (LP pellets). In addition, 6 g of Yb_2_O_3_ powder was taken and pressed into a plate with a thickness of approximately 2 mm to provide support for the SP and LP pellets.

### 2.2. Fabrication of S-D Y-123 Bulks

**Figure 1:**
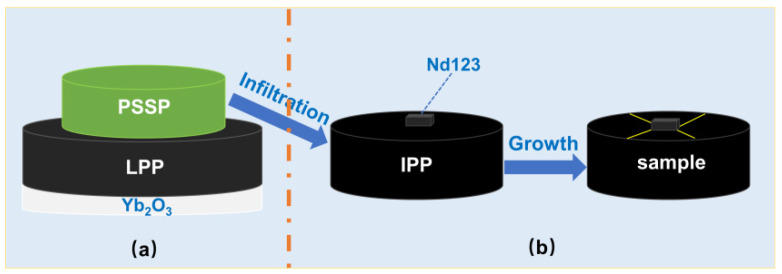
Illustration of the entire heat treatment process. Among the images, (**a**) is the infiltration process and (**b**) is the growth process.

Generally, the maximum temperature during the heat treatment is limited to approximately 1050 °C–1060 °C while using the traditional TSMG or TSIG process for the preparation of S-D Y-123 bulks if Nd-123 crystal seeds are utilized. However, the infiltration of the liquid phase at higher temperatures helps to optimize the microstructure of the sample [23]. To identify the optimum temperature for liquid phase infiltration, we employed a novel method that divided the RE + 011 IG process into two stages: the IS and the GS, as illustrated in Figure 1. The IS process was carried out at various temperatures from 1000 °C–1300 °C in this study.

**Figure 2:**
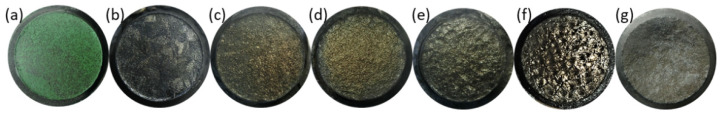
Top surface morphology of the IP pellets after IS. (**a**) Ti = 1000 °C, labeled as IP1, (**b**) 1050 °C, IP2, (**c**) 1100 °C, IP3, (**d**) 1150 °C, IP4, (**e**) 1200 °C, IP5, (**f**) 1250 °C, IP6, and (**g**) 1300 °C, IP7.

During the IS process, the samples were heated to 910 °C and maintained for a duration of 10 h to convert the mixed powder of (Y_2_O_3_ + BaCuO_2_) into Y-211 and to optimize the particle size of Y-211 and the porosity of the SP pellets, while also enhancing the mechanical rigidity of the precursor pellets [27]. Subsequently, the temperature was raised to the chosen levels (i.e., 1000, 1050, 1100, 1150, 1200, 1250, and 1300 °C) at a rate of 100 °C·h^−1^ and maintained for 1 h to ensure the complete infiltration of the liquid phase into the SP pellet. The sample obtained after the IS will be referred to as the IP pellet, and the top surface of these pellets are shown in Figure 2.

During the GS process, an Nd-123 seed was carefully placed on the IP pellet’s top surface, and the ab-plane of the crystal seed was aligned parallel to the surface, as shown in Figure 1b. Next, the arrangement was heated to 1045 °C and held at this temperature for 1.5 h, and it was cooled down to 1008 °C rapidly. Subsequently, it was more slowly cooled at a rate of 0.3 °C·h^−1^ to 988 °C before the furnace was allowed to cool down to room temperature. Lastly, all of the S-D Y-123 samples were annealed in a temperature range of 450 °C–410 °C in flowing oxygen for approximately 200 h.

### 2.3. Measurements

Levitation forces: The levitation forces of the sample were measured in the zero-field-cooled state at 77 K (liquid nitrogen temperature) using a home-made device [28]. Additionally, an Nd-Fe-B permanent magnet of the same dimensions (φ = 20 mm) as the sample was employed, which has a surface magnetic field of approximately 0.5 T [29]. It is essential to note that the seed crystals were removed from the top surface, and the samples were polished to a flat finish prior to testing the levitation force on each sample.

Microstructure: The microstructure of the IP pellets and the final Y-123 bulks were observed using scanning electron microscopy (SEM) (HITACHI, SU8220).

## 3. Results and Discussion

### 3.1. Surface Morphology of the IP Pellet

Figure 2 shows the top surface morphology of the IP pellets after the IS. It can be seen that there are significant differences in the color and morphology of the top surface in the different IP pellets. For example, in pellet IP1, most of the surface of the sample showed a green color, indicating that at this temperature, the liquid phase has not sufficiently infiltrated into the SP pellet, as shown in Figure 2a. From pellets IP2–IP6, no crystal seeds were employed, which resulted in the random nucleation of individual Y-123 sub-grains. However, as the T*_i_* increased, there was a significant difference in the gloss, and the surface of the IP pellet became rougher and rougher, as shown in Figure 2b–f. In pellet IP7, at a temperature of 1300 °C, it did not maintain the original cylindrical shape like the other six samples but instead appeared to be severely concave in the center of its surface, as shown in Figure 2g. This may have been caused by the temperature being too high, causing decomposition of the Y-211 phase in the air atmosphere (mp. approximately 1277 °C) [30], leading to the absence of the Y-211 phase in the Y-123 matrix, thus failing to maintain the original shape of the solid-phase preform. Therefore, we can confirm that the effect of the T*_i_* is not negligible, and there exists an optimal temperature.

**Figure 3:**
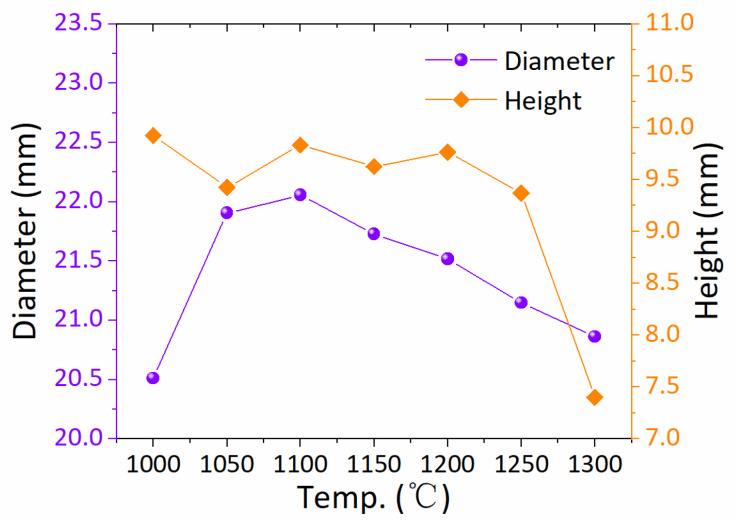
The correlation between the dimensions of the IP pellets and the T*_i_*. The left Y-axis correlates with the diameter of the IP pellets, and the right Y-axis correlates with the thickness of the IP pellets.

In Figure 3, the diameter and height of the IP pellets are graphically represented as a function of the T*_i_*. The figure shows that the diameter of the IP pellet initially increases and then decreases with the rise of the T*_i_*, and the pellet IP3 has the largest diameter. On the other hand, the height of the infiltration pellet shows an overall decreasing trend with an increasing T*_i_*, with IP3 showing the largest height except for IP1. This trend of change is similar to the findings of previous studies [21,31]. The possible reason for the above variation is that the liquid phase infiltration occurs at higher temperatures, and gas continues to escape from within the SP pellets. Therefore, the T*_i_* is a crucial factor when the TSIG method is employed to fabricate REBCO bulk superconductors.

### 3.2. Surface Morphology of the S-D Y-123

**Figure 4:**
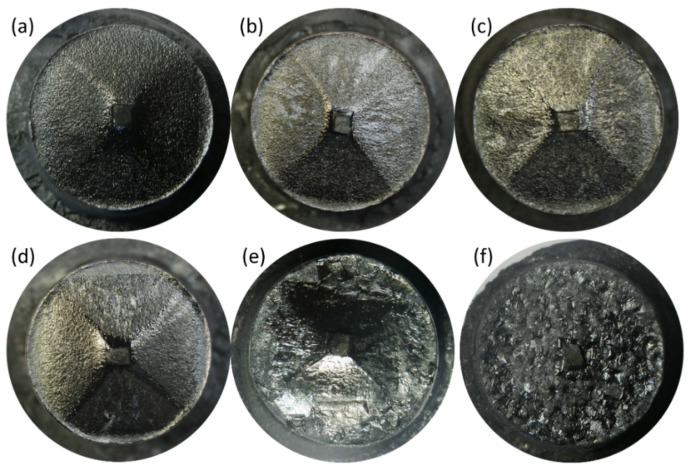
The top views of the final Y-123 bulks. (**a**) Sample S1, fabricated from IP1, (**b**) S2, IP2, (**c**) S3, IP3, (**d**) S4, IP4, (**e**) S5, IP5, (**f**) S6, IP6.

Figure 4 presents the top view of the S-D Y-123 bulks that were grown by using the modified RE + 011 IG process. It should be added that due to the failure of IP7, no sample of its counterpart was grown in this study. As can be seen from these figures, the Y-123 bulks were epitaxially nucleated and grown from Nd-123 seeds for all samples; however, the grown crystals were significantly different for each sample, especially in the samples S5 and S6. In other words, the samples S1-S4 exhibited a similar morphological characteristic with X-shaped facet lines, indicating that the Y-123 crystals formed S-D Y-123 grains, but the grains in the sample S5 had not yet completely grown to the edge of the entire bulk; in contrast, the area of the S-D region formed in sample S6 was slightly larger than that of the seed crystal. This indicates that the failure of growth in this sample was not caused by a failure of the seed crystal. Possible reasons for the growth failure of samples S5 and S6 will be analyzed in relation to the SEM–EDS elemental mappings of IP pellets.

### 3.3. Levitation Force

**Figure 5:**
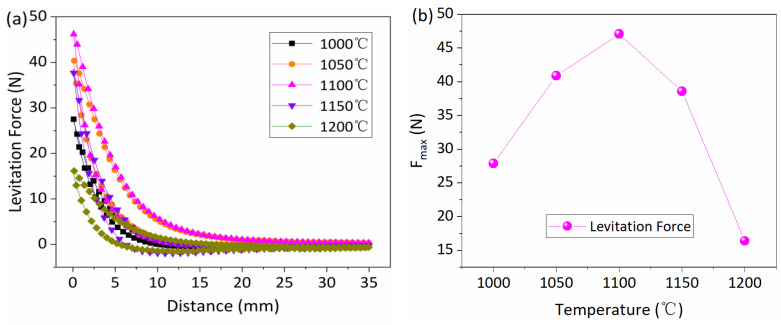
(**a**) Levitation force as a function of distance at 77 K for the S-D Y-123 bulks. (**b**) Collecting the F_max_ of the sample obtained under different T_i_ conditions and representing the correlation between the T*_i_* and the F_max_.

The magnetic performances of the Y-123 bulks are linked to the V_f_ of the grown S-D region. However, the S-D region of S6 is extremely small in the aforementioned sample; therefore, only the levitation force of samples S1–S5 was measured at 77 K. In the testing process, the maximum levitation force (F_max_) of the sample was measured at a distance of 0.5 mm between the two closest surfaces of the permanent magnet and the Y-123 bulks; the results are shown in Figure 5a. At the same time, the F_max_ values for the five samples were collected and are illustrated in Figure 5b. It can be observed that the F_max_ value initially increased and then decreased with the rise of the T*_i_*. Among the five samples, the F_max_ value reached 47.1 N, which was obtained with sample S3 (T*_i_* = 1100 °C). This exhibited a relatively high magnetic levitation force in comparison to other works reported in the literature [31,32,33].

### 3.4. Microstructure

#### 3.4.1. SEM–EDS Mapping of IP Pellet

**Figure 6:**
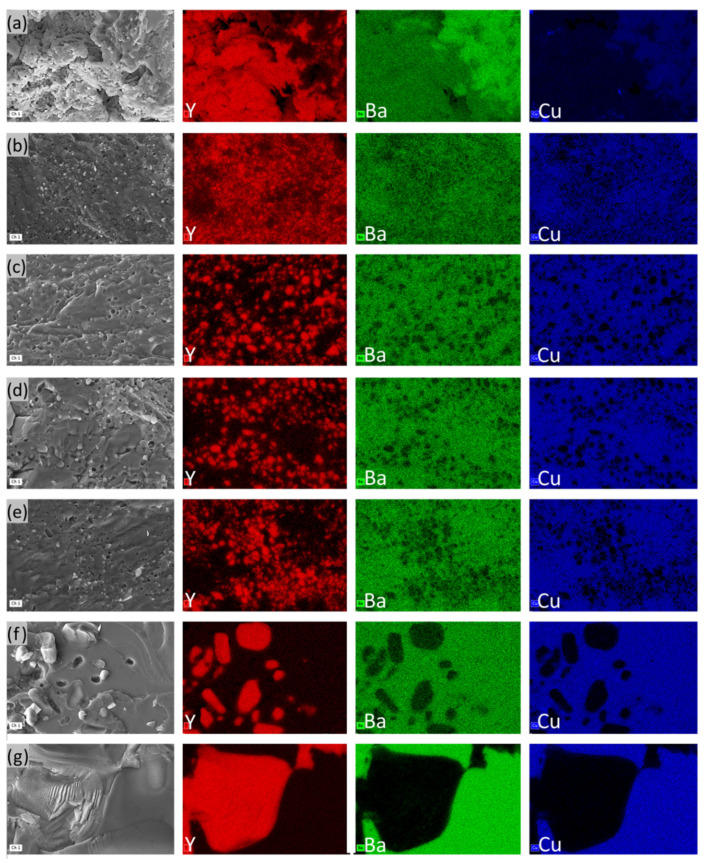
SEM–EDS elemental mapping of IPP pellets at 3000 magnification with an acceleration voltage of 20 kV. (**a**) IP1, (**b**) IP2, (**c**) IP3, (**d**) IP4, (**e**) IP5, (**f**) IP6, and (**g**) IP7, showing the Y (red), Ba (green), and Cu (blue).

SEM and EDS elemental mapping were used to characterize and analyze the microscopic morphology of the IP pellets, which are presented in Figure 6. As can be seen in the distribution of Y elements in these images, there is a significant difference in each sample. Particularly from the pellets IP4–IP7 (infiltrated at 1150 °C–1300 °C), it begins to appear unevenly distributed; agglomerations of Y elements are shown in Figure 6 d–g.

As we know, the yttrium ions (Y^3+^) for the peritectic solidification reaction, which are only provided by the decomposition of the Y-211 phase [34], and the larger size of the Y-211 particle denote the lower Y-211 dissolution [35]. In other words, the crystal growth of the Y-123 phase is determined by the concentration gradient diffusion of the Y^3+^ ions dissolved from the Y-211 phase in the presence of the Ba–Cu–O liquid phases [36], and the large Y-211 particles severely disturb the homogeneous growth of the Y-123 crystal [37]. This may be one of the possible reasons for the failure of the samples in Figure 4e, f.

#### 3.4.2. Y-211 Particle Distribution of Final Sample

**Figure 7:**
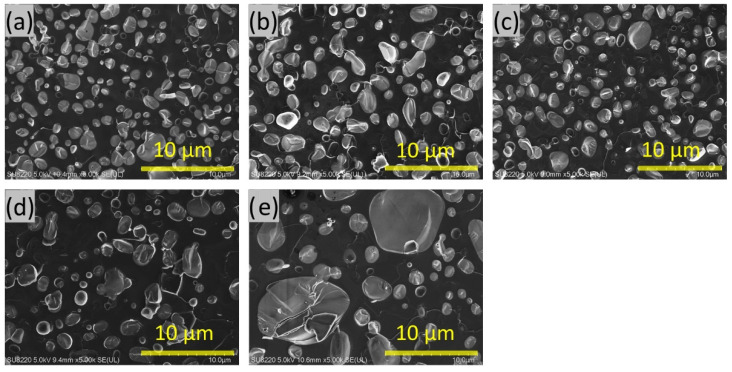
SEM images of the Y-123 bulks. (**a**) S1, (**b**) S2, (**c**) S3, (**d**) S4, (**e**) S5.

Figure 7 illustrates the microstructure of the S-D Y-123 samples, which is associated with their physical performances. The software Nano Measure (V1.2.0) was utilized to analyze the average size of the Y-211 particles embedded within the Y-123 matrix. The SEM measurements were conducted using a small crystal that naturally cleaved 4 mm below the Nd-123 seed crystal. The images indicate a nearly uniform distribution of Y-211 particles in samples S1 to S3, and their average size was approximately 1.2 ± 0.1 µm. On the contrary, the distribution of the Y-211 particles embedded in the Y-123 phase was not uniform in samples S4 and S5, and their average sizes were approximately 1.7 µm and 2.9 µm, respectively. Moreover, the V_f_ of the Y-211 phase in the Y-123 matrix for S1 to S5 was 40.8%, 37.2%, 32.7%, 30.5%, and 46.4%, respectively, as shown in Table 1.

For the rise of the Y-211 content in sample S1 (40.8%, T*_i_* = 1000 °C), the possible reason is that low temperatures cause poor fluidity of the liquid phase, resulting in insufficient liquid phase infiltration into the SP pellet. On the contrary, for sample S5 (46.3%, T*_i_* = 1200 °C), an excessive T*_i_* may lead to the loss in the liquid phase during the IS. It is suggested that the particle sizes of Y-211 and its V_f_ in the Y-123 phases may be optimized by high-temperature infiltration at optimal temperatures.

As we know, during the process of peritectic solidification, the smaller, unreacted Y-211 particles are commonly entrapped within the Y-123 phase. This leads to the formation of the Y-211/Y-123 interface, which is generally recognized as the flux pinning center [38]. Thus, for a specified V_f_ of Y-211 particles, the interface region between Y-123 and Y-211 is substantial when the size of the Y-211 particles is small. In addition, according to previous research results, the V_f_ of the Y-211 phase of approximately 30% is the best content [39]. Therefore, in these samples, the inferior properties of sample S1 may be caused by the relatively large Y-211 content [11]. In contrast, for sample S5, they could be due to the combination of large Y-211 particle sizes and a high V_f_ within the sample. This may be because sample S5 was subjected to a maximum temperature of 1200 °C for 1 h. At such high temperatures, the Y-211 secondary phase begins to decompose into Y_2_O_3_ and a liquid phase, while larger Y-211 particles remain in the sample, which adversely affects its flux pinning effect.

## 4. Conclusions

Microstructure of the S-D Y-123 bulks can be optimized via high-temperature infiltration of the liquid phase, however, the conventional TSMG and TSIG methods are not suitable for this purpose due to the melting point constraints of the seed crystal. In this work, a group of S-D Y-123 bulks was prepared by a modified Y + 011 IG technique after high-temperature infiltration (1000 °C–1300 °C). At the same time, the relationship between the T*_i_*, the surface morphology, the microstructure of infiltrated pellets, and the well-grown final samples, as well as the physical properties has been investigated in detail. The following was found:

(1) The distribution, average particle size, and V_f_ of the Y-211 phase in the sample were significantly affected by the T*_i_*.

(2) When the T*_i_* ranged from 1000 to 1150 °C, it was possible to prepare Y-123 bulk samples with an S-D morphology; however, when the T*_i_* reached approximately 1200 °C or higher, there was a noticeable decrease in the area of the S-D region in the final Y-123 samples.

(3) The levitation forces of the S-D Y-123 bulks initially increased and then decreased with the rise of the T*_i_*. The largest levitation force recorded was 47.1 N (at 77 K, 0.5 T), and the corresponding sample was infiltrated at a temperature of 1100 °C.

## Figures and Tables

**Table 1:** The volume fraction and average particle size (AVG) of Y-211 in different Y-123 samples.

Final Sample	Infiltration Temp.	V_f_ of Y-211	AVG of Y-211
S1	1000 °C	40.8%	1.16 µm
S2	1050 °C	37.2%	1.35 µm
S3	1100 °C	32.7%	1.11 µm
S4	1150 °C	30.5%	1.67 µm
S5	1200 °C	46.4%	2.09 µm

## Data Availability

All data generated or analyzed during this study are included in this study.
